# Integrative taxonomy of Tetraplosphaeriaceae from the Nujiang River Basin, China, reveals two new species and a new record

**DOI:** 10.3897/mycokeys.134.194692

**Published:** 2026-06-18

**Authors:** Xue-Ling Ma, Ming-Jun Zhu, Feng-Xiang Ma, Mei-Rui Chen, Zong-Long Luo, Hong-Wei Shen

**Affiliations:** 1 College of Agriculture and Biological Science, Dali University, Dali 671003, Yunnan, China College of Agriculture and Biological Science, Dali University Dali China; 2 Co-Innovation Center for Cangshan Mountain and Erhai Lake Integrated Protection and Green Development of Yunnan Province, Dali University, Dali 671003, Yunnan, China Co-Innovation Center for Cangshan Mountain and Erhai Lake Integrated Protection and Green Development of Yunnan Province, Dali University Dali China

**Keywords:** Lignicolous freshwater fungi, morphology, phylogeny, taxonomy, two new species

## Abstract

Lignicolous freshwater fungi play crucial ecological roles in nutrient cycling and wood decomposition, yet their diversity remains insufficiently documented in many regions. In this study, we conducted an integrative taxonomic and phylogenetic investigation of Tetraplosphaeriaceae from submerged woody substrates in the Nujiang River Basin, Yunnan Province, China. Five taxa were identified, comprising two new species, *Aquatisphaeria fusispora* and *Tetraploa nujiangensis*, one new record for China, *T. thailandica*, and two previously described species, *A. thailandica* and *T. yunnanensis*. Detailed morphological observations were complemented by multigene phylogenetic analyses based on ITS, LSU, SSU, and *tub*2 sequences data, which robustly supported their placement within Tetraplosphaeriaceae. The introduction of new taxa and the discovery of novel geographic record have not only expanded the known species diversity of this family but also extended its documented distribution range. Our findings highlight the Nujiang River Basin as an underexplored reservoir of lignicolous freshwater fungal diversity and provide a solid foundation for future systematic, biogeographic, and ecological studies of freshwater fungi in subtropical Asia.

## Introduction

Lignicolous freshwater fungi are a specialized group of fungi inhabiting freshwater environments, including rivers, lakes, ponds, and wetlands, where they colonize submerged woody substrates (Hyde et al. 2016; Luo et al. 2018). As primary decomposers of woody organic matter, they play essential roles in decomposition processes, nutrient cycling, and energy transfer within freshwater ecosystems, thereby contributing significantly to ecosystem functioning (Shearer 1993; Wong et al. 1998; Luo et al. 2004; Hyde et al. 2016). Although considerable progress has been made in documenting their diversity over recent decades, the taxonomy and evolutionary relationships of many lignicolous freshwater fungal groups remain insufficiently resolved, highlighting the need for continued systematic and integrative studies.

Tetraplosphaeriaceae (Pleosporales, Dothideomycetes) was established by Tanaka et al. (2009) to accommodate *Polyplosphaeria*, *Pseudotetraploa*, *Quadricrura*, *Tetraplosphaeria*, and *Triplosphaeria*, with *Tetraplosphaeria* designated as the type genus. *Tetraplosphaeria* was subsequently treated as a synonym of *Tetraploa* (Hyde et al. 2013), consequently, *Tetraploa* is currently recognized as the type genus of Tetraplosphaeriaceae (Tang et al. 2023). The sexual morphs of Tetraplosphaeriaceae are characterized by immersed to superficial ascomata, cylindrical to clavate, short pedicellate asci, and narrowly fusiform to broadly cylindrical, septate, hyaline to pale brown ascospores (Tanaka et al. 2009; Hyde et al. 2013). The asexual morphs are tetraploa-like hyphomycetes, characterized by monoblastic conidiogenous cells, short cylindrical to obpyriform conidia, composed of several columns at the conidial base with several setose appendages at the apex (Tanaka et al. 2009; Hyde et al. 2013). Based on morphological and molecular evidence (ITS, LSU, and SSU), Ariyawansa et al. (2015) transferred *Shrungabeeja* to Tetraplosphaeriaceae. Subsequently, *Aquatisphaeria*, *Byssolophis*, *Ernakulamia* and *Pseudopolyplosphaeria* were incorporated into this family (Delgado et al. 2017; Pem et al. 2019; Li et al. 2021; Zhang et al. 2023). Recently, He et al. (2026) introduced additional genus, *Neotriplosphaeria*, increasing the total number of genera in Tetraplosphaeriaceae to eleven. To date, members of this family are primarily occurred as saprobes or pathogens on bamboo plants and unidentified decayed woody substrates, inhabiting both aquatic and terrestrial habitats (Tanaka et al. 2009; Hyde et al. 2013; Hongsanan et al. 2020; Li et al. 2021; Yu et al. 2022).

Yunnan Province is in the southwest of China; it is a low-latitude, high-altitude inland province, and is one of the biodiversity hotspots in the Yunnan–Guizhou Plateau (Xu et al. 2017). Currently, more than 400 species of lignicolous freshwater fungi have been reported from Yunnan (Shen et al. 2022, 2023, 2024, 2025; Wang et al. 2024). These fungi mainly belong to the classes Dothideomycetes and Sordariomycetes of the phylum Ascomycota, with a few species belonging to the classes Eurotiomycetes and Leotiomycetes (Shen et al. 2022, 2025). As one of the major international river systems in Yunnan Province, the Nujiang River Basin extends from north to south across multiple climatic zones, exhibiting remarkable variations in hydrological conditions and ecological environments (Zhang et al. 2024). Such environmental heterogeneity provides diverse ecological niches and evolutionary drivers for freshwater fungi. The wide range of available substrates in this region, including driftwood, submerged wood, and bamboo debris, offers ideal habitats for the growth and evolution of lignicolous freshwater fungi (Luo et al. 2017, 2018, 2019; Bao et al. 2021), particularly members of the family Tetraplosphaeriaceae (Tanaka et al. 2009; Hyde et al. 2013; Hongsanan et al. 2020; Yu et al. 2022). However, to date, no comprehensive and systematic investigation of lignicolous freshwater fungi has been conducted in the Nujiang River Basin, and our current understanding of their diversity, distribution patterns, and ecological roles in this region remains fragmentary and insufficient.

During our investigation of lignicolous freshwater fungi in the Nujiang River Basin, seven strains of Tetraplosphaeriaceae were collected. Based on detailed morphological examination and multigene phylogenetic analysis, we introduce two new species, *Aquatisphaeria fusispora* and *Tetraploa nujiangensis*, report *T. thailandica* as a new record for China, and re-examine two previously species, *A. thailandica* and *T. yunnanensis*, all of which are described and illustrated. In addition, one new combination is proposed, *A. compacta* (≡ *Ceratosporella compacta*). These findings enrich the current knowledge of Tetraplosphaeriaceae and lignicolous freshwater fungi in China, particularly within the Nujiang River Basin, and contribute to a more comprehensive understanding of their taxonomic diversity and regional distribution patterns. The results also provide a scientific basis for future biodiversity assessments, conservation strategies, and sustainable utilization of freshwater fungal resources in this ecologically important river system.

## Materials and methods

### Sample collection, specimen examination and isolation

Submerged decaying woods were collected from Nujiang River (including tributaries and main streams) in Yunnan Province, China. The samples were brought to the laboratory in ziplock plastic bags and incubated in plastic boxes lined with moistened tissue paper at room temperature for one week. The morphological structures were observed as described in Luo et al. (2018) and Shen et al. (2023).

Fungal colonies on natural substrates were observed using a Guiguang GL-99BI compound stereomicroscope (Guilin Guiguang Instrument Co., Ltd., Guilin, China) and then photographed with a Nikon SMZ1000 stereo zoom microscope (NIKON CORPORATION, Tokyo, Japan). Fungal structures were photographed using a Nikon ECLIPSE Ni-U compound microscope (NIKON CORPORATION, Tokyo, Japan) fitted with a Nikon DS-Ri2 digital camera (NIKON CORPORATION, Tokyo, Japan), as per the guidelines provided by Luo et al. (2018). Single spore isolation was conducted by following the methods described by Senanayake et al. (2020). Measurements were made with the Tarosoft (R) Image Frame Work program, and photo plates representing fungal structures were processed in Adobe Photoshop CS5 software (Adobe Systems Inc., San Jose, CA, USA). Herbarium specimens (dry woody branches with fungal material) were deposited in the herbarium of Cryptogams, Kunming Institute of Botany Academia Sinica (HKAS), Kunming, China. The isolates obtained in this study were deposited in the China General Microbiological Culture Collection Center (CGMCC), Beijing, China, and the Kunming Institute of Botany Culture Collection Center (KUNCC), Kunming, China. Fungal Names number (FN) of the new species was registered (https://nmdc.cn/fungalnames/).

### DNA extraction, PCR amplification, and Sequencing

DNA extraction, PCR amplification, sequencing, and phylogenetic analysis were done following the methods of Senanayake et al. (2020). Mycelia for DNA extraction from each isolate was grown on PDA for 3–4 weeks at 24 °C. Total genomic DNA was extracted from 100–300 mg axenic mycelium, scraped from the edges of the growing culture, using a sterile scalpel, and transferred to a 1.5 mL microcentrifuge tube using sterilized inoculum needles. Mycelium was ground to a fine powder with liquid nitrogen or quartz sand to break the cells for DNA extraction. DNA was extracted with the Fungal gDNA Isolation Kit (B08WB206A-50) following manufacturer guidelines. Four gene regions, ITS, LSU, SSU, and *tub*2 were amplified using ITS5/ITS4 (White et al. 1990), LR0R/LR5 (Vilgalys and Hester 1990), NS1/NS4 (Liu et al. 1999), and T1/BT2b (Glass and Donaldson 1995) primer pairs, respectively. Primer sequences are available in the WASABI database on the AFTOL website (aftol.org). The amplifications were performed in a 25 μL reaction volume containing 9.5 μL ddH2O, 12.5 μL 2 × Taq PCR Master Mix with blue dye (Shanghai Sangon Biological Engineering Technology and Services Co., Shanghai, China), 1 μL DNA template, and 1 μL of each primer (10 μM). PCR products were checked on 1% agarose electrophoresis gels stained with GelRed (Beijing TsingKe Biotech Co., Ltd., Beijing, China). The sequencing reactions were carried out using the primers mentioned above by Shanghai Sangon Biological Engineering Technology and Services Co., Shanghai, China.

### Phylogenetic analyses

The Basic Local Alignment Search Tool (BLAST) searches in the National Center of Biotechnology Information (NCBI) preliminarily screens out strains of Tetraplosphaeriaceae. Four gene markers, LSU, ITS, SSU, and *tub*2, were used for the multigene analyses, with the whole or part of them concatenated for different fungal groups. Singlelocus sequences were aligned using the online multiple alignment program MAFFT version 7 (Rozewicki et al. 2019), and this alignment was manually optimized in BioEdit version 7.0.5.3 (Hall 1999). The concatenated sequence alignments were obtained from SequenceMatrix version 1.7.8 (Vaidya et al. 2011). Maximum likelihood (ML) analysis was performed using Randomized Axelerated Maximum Likelihood High-Performance Computing 2 (RAxML-HPC2) on ACCESS (Stamatakis 2006; Stamatakis et al. 2008) on the Extreme Science and Engineering Discovery Environment (XSEDE) TeraGrid of the CIPRES Science Gateway online platform (Miller et al. 2010) with rapid bootstrap analysis, which was followed by 1,000 bootstrap replicates. The final tree was selected among the suboptimal trees from each run by comparing the likelihood scores under the general time-reversible gamma (GTRGAMMA) parameter substitution model.

Bayesian inference (BI) analysis was performed in a likelihood framework implemented in MrBayes version 3.1.2 (Ronquist et al. 2012). The Markov Chain Monte Carlo (MCMC) sampling approach was used to calculate posterior probabilities (PP) (Ranala and Yang 1996). A Bayesian analysis of six simultaneous Markov chains was run for 10,000,000 generations, with trees sampled at intervals of every 1,000 generations. The sequences generated in this study have been deposited in GenBank and are listed in Table 1.

**Table 1.** Isolates and sequences were in this study; the newly generated sequences are indicated in cells with light grey shading, and the type strains are indicated in bold. “–” stands for no sequence data in GenBank.

**Taxon**	**Voucher/Strain Number**	**GenBank Accession Number**			
		**SSU**	**ITS**	**LSU**	***tub*2**
***Aquatisphaeria bambusae***	**ZHKUCC 24–0007**	**PP336663**	**PP336659**	**PP336667**	**PP346805**
***Aquatisphaeria fusispora***	**CGMCC 3.29449**	**PX607356**	**PX463767**	**PX607352**	**PX828504**
***Aquatisphaeria thailandica***	**MFLUCC 21–0025**	**MW890967**	**MW890969**	**MW890763**	**–**
*Aquatisphaeria thailandica*	ZHKUCC 24–0005	PP336662	PP336657	PP336665	PP346803
*Aquatisphaeria thailandica*	CGMCC 3.29448	–	PX607345	PX607350	–
***Ernakulamia americana***	**CBS 146616**	**–**	**PV809787**	**–**	**PV742390**
***Ernakulamia cochinensis***	**MFLUCC 18–1237**	**MT864326**	**MT627670**	**MN913716**	**–**
***Ernakulamia krabiensis***	**KUMCC 18–0240**	**MK347880**	**MK347773**	**MK347990**	**–**
***Ernakulamia syagri***	**CCMB 742**	**PQ726987**	**PP889895**	**PP889896**	**–**
***Ernakulamia tanakae***	**NFCCI 4615**	**–**	**MN937229**	**MN937211**	**MN938312**
***Ernakulamia xishuangbannaensis***	**KUMCC 17–0187**	**MH260354**	**MH275080**	**MH260314**	**–**
***Montanitestudina hydei***	**SQUCC 15173**	**MW077165**	**MW077149**	**MW077158**	**–**
***Muritestudina chiangraiensis***	**MFLUCC 17–2551**	**MG602249**	**MG602247**	**MG602248**	**–**
***Neotriplosphaeria yadongensis***	**HKAS 144527**	**PQ675371**	**PQ684991**	**PQ675410**	**–**
*Neotriplosphaeria yadongensis*	HKAS 144528	PQ675372	PQ684992	PQ675411	–
***Parapolyplosphaeria thailandica***	**MFLU 15–3273**	**–**	**KU248766**	**KU248767**	**–**
***Polyplosphaeria fusca***	**KT 1616**	**AB524463**	**AB524789**	**AB524604**	**AB524851**
*Polyplosphaeria fusca*	KT 1640	AB524464	AB524790	AB524605	AB524852
***Polyplosphaeria guizhouensis***	**GZCC 23–0598**	**–**	**OR427327**	**OR438888**	**OR449118**
***Polyplosphaeria hainanensis***	**GZCC 23–0599**	**OR438285**	**OR427323**	**OR438889**	**OR449115**
***Polyplosphaeria nabanheensis***	**KUMCC 16–0151**	**MH260352**	**MH275078**	**MH260312**	**MH412745**
***Polyplosphaeria nigrospora***	**ZHKUCC 22–0132**	**–**	**OR164935**	**OR164963**	**–**
***Polyplosphaeria pandanicola***	**KUMCC 17–0180**	**MH260353**	**MH275079**	**MH260313**	**–**
***Pseudotetraploa aquatica***	**KUNCC 23–13375**	**–**	**PQ340473**	**PP189908**	**PQ456973**
***Pseudotetraploa bambusicola***	**CGMCC 3.20939**	**ON332923**	**ON332915**	**ON332933**	**–**
*Pseudotetraploa bambusicola*	UESTCC 22–0005	ON332924	ON332916	ON332934	–
***Pseudotetraploa curviappendiculata***	**HHUF 28582**	**AB524467**	**AB524792**	**AB524608**	**AB524854**
*Pseudotetraploa curviappendiculata*	HHUF 28590	AB524468	AB524793	AB524609	AB524855
*Pseudotetraploa javanica*	HHUF 28596	AB524470	AB524795	AB524611	AB524857
***Pseudotetraploa longissima***	**HHUF 28580**	**AB524471**	**AB524796**	**AB524612**	**AB524858**
***Pseudotetraploa phyllostachydisa***	**ZHKUCC 24–0006**	**–**	**PP336658**	**PP336666**	**PP346804**
***Pseudotetraploa rajmachiensis***	**NFCCI 4618**	**–**	**MN937222**	**MN937204**	**MN938305**
***Pseudotetraploa yangjiangensis***	**ZHKUCC 24–0008**	**–**	**PP336660**	**PP336668**	**PP346806**
***Pseudotetraploa yunnanensis***	**KUNCC 10464**	**–**	**OR449073**	**OR438891**	**–**
***Quadricrura bicornis***	**CBS 125427**	**AB524472**	**AB524797**	**AB524613**	**AB524859**
***Quadricrura meridionalis***	**CBS 125684**	**AB524473**	**AB524798**	**AB524614**	**AB524860**
***Quadricrura septentrionalis***	**CBS 125430**	**AB524475**	**AB524800**	**AB524616**	**AB524862**
*Quadricrura septentrionalis*	CBS 125428	AB524476	AB524801	AB524617	AB524863
***Shrungabeeja aquatica***	**MFLUCC 18–0664**	**–**	**MT627722**	**MT627663**	**–**
***Shrungabeeja fluviatilis***	**GZCC 20-0505**	**OP377989**	**OP377804**	**OP377903**	**–**
***Shrungabeeja longiappendiculata***	**BCC76463**	**KT376471**	**KT376474**	**KT376472**	**–**
*Shrungabeeja longiappendiculata*	BCC 76464	–	KT376475	KT376473	–
*Shrungabeeja vadirajensis*	MFLUCC 17–2362	–	MT627681	MN913685	–
***Tetraploa aquatica***	**MFLU 19–0995**	**–**	**MT530448**	**MT530452**	**–**
*Tetraploa aristata*	CBS 996.70	AB524486	AB524805	AB524627	AB524867
***Tetraploa bambusae***	**KUMCC 21–0844**	**ON077073**	**ON077078**	**ON077067**	**ON075065**
***Tetraploa cylindrica***	**KUMCC 20–0205**	**ON555690**	**ON555689**	**MT893204**	**ON564477**
***Tetraploa dashaoensis***	**KUMCC 21–0010**	**OL473556**	**OL473549**	**OL473555**	**OL505601**
***Tetraploa dwibahubeeja***	**NFCCI 4621**	**–**	**MN937226**	**MN937208**	**MN938309**
***Tetraploa endophytica***	**CBS 147114**	**–**	**KT270279**	**MW659165**	**–**
***Tetraploa guizhouensis***	**GZCC 25–0694**	**–**	**PV982846**	**–**	**PV979721**
***Tetraploa hainanensis***	**GZCC 23–0601**	**OR438286**	**OR427325**	**OR438892**	**OR449116**
*Tetraploa juncicola*	CBS 149046	–	ON603780	ON603800	–
***Tetraploa lignicola***	**KUNCC 10794**	**ON422300**	**ON422286**	**ON422294**	**–**
***Tetraploa linzhiensis***	**HKAS 144535**	**PQ675373**	**PQ684993**	**PQ675412**	**–**
***Tetraploa longiappendiculata***	**KUNCC 23–13397**	**OR743218**	**OR589327**	**OR600975**	**–**
***Tetraploa maritima***	**MFLU 24–0455**	**PQ778940**	**PQ778934**	**PQ778930**	**PQ885482**
***Tetraploa nagasakiensis***	**KT 1682**	**AB524489**	**AB524806**	**AB524630**	**AB524868**
***Tetraploa nanpanjiangensis***	**GZCC 25–0695**	**–**	**PV982847**	**PV982827**	**PV979722**
***Tetraploa nujiangensis***	**CGMCC 3.29451**	**PX607354**	**PX463798**	**PX607349**	**–**
*Tetraploa nujiangensis*	KUNCC 25–19350	PX607355	PX463769	PX607348	–
***Tetraploa obpyriformis***	**KUMCC 21–0011**	**OL473557**	**OL473558**	**OL473554**	**OL505600**
***Tetraploa oryzae***	**MFLU 23–0195**	**OR458352**	**OR438372**	**OR438841**	**–**
***Tetraploa palmae***	**HKAS 115638**	**NG245369**	**–**	**NG245594**	**–**
***Tetraploa phoenicis***	**HKAS 115675**	**PP639246**	**PP592486**	**PP621117**	**–**
***Tetraploa pseudoaristata***	**NFCCI 4624**	**–**	**MN937232**	**MN937214**	**MN938315**
***Tetraploa puzheheiensis***	**KUMCC 20–0151**	**–**	**MT627744**	**MT627655**	**–**
***Tetraploa sasicola***	**KT 563**	**AB524490**	**AB524807**	**AB524631**	**AB524869**
*Tetraploa scheueri*	CY 112	–	HQ607964	–	–
*Tetraploa* sp.	HKAS 131566	–	PP768923	PP768935	–
***Tetraploa submersa***	**ZHKUCC 24–0009**	**PP336664**	**PP336661**	**PP336669**	**PP346807**
***Tetraploa thailandica***	**MFLUCC 21–0030**	**MZ413274**	**MZ412518**	**MZ412530**	**–**
*Tetraploa thailandica*	CGMCC 3.29453	**–**	**PX506027**	**PX506073**	**–**
***Tetraploa thrayabahubeeja***	**NFCCI 4627**	**–**	**MN937235**	**MN937217**	**MN938318**
***Tetraploa verrucispora***	**GZCC 25–0696**	**PV982862**	**PV982845**	**PV982826**	**PV979720**
***Tetraploa verrucosa***	**KUNCC 23–13151**	**PX218444**	**PQ845967**	**PV536416**	**–**
***Tetraploa wurfbainiae***	**ZHKUCC 23–0954**	**OR626043**	**OR626039**	**OR626041**	**OR653395**
***Tetraploa yakushimensis***	**KT 1906**	**AB524491**	**AB524808**	**AB524632**	**AB524870**
***Tetraploa yunnanensis***	**MFLUCC 19–0319**	**MT864341**	**MT627743**	**MN913735**	**–**
*Tetraploa yunnanensis*	CGMCC 3.29454	PX607357	PX607347	PX607353	PX828505
*Tetraploa yunnanensis*	CGMCC 3.29455	PX607358	PX607346	PX607351	PX828506
***Triplosphaeria acuta***	**KT 1170**	**AB524492**	**AB524809**	**AB524633**	**AB524871**
***Triplosphaeria bambusicola***	**CGMCC 3.25610**	**–**	**PQ067805**	**PQ067721**	**–**
***Triplosphaeria cylindrica***	**KT 1800**	**AB524494**	**AB524810**	**AB524635**	**AB524872**
***Triplosphaeria fusiformis***	**CGMCC 3.25609**	**PQ066560**	**PQ067804**	**PQ067720**	**–**
***Triplosphaeria guizhouensis***	**GZCC 19–0512**	**MW134632**	**OQ646060**	**MW133854**	**OQ659019**
***Triplosphaeria maxima***	**KT 870**	**AB524496**	**AB524812**	**AB524637**	**AB524874**
*Triplosphaeria* sp.	HHUF 27481	AB524499	AB524815	AB524640	AB524877
***Triplosphaeria yezoensis***	**CBS 125436**	**AB524497**	**AB524813**	**AB524638**	**AB524875**
***Triplosphaeria yunnanensis***	**KUNCC 23–13443**	**PQ435268**	**PQ340474**	**PP189912**	**PQ456974**

## Result

### Phylogenetic analyses

Phylogram generated from maximum likelihood analysis based on combined LSU, ITS, SSU and *tub*2 sequence dataset representing Tetraplosphaeriaceae. Eighty-seven strains are included in the combined analyses which comprise 3000 characters including gaps (847 characters for LSU, 523 characters for ITS, 991 characters for SSU, 639 characters for *tub*2). *Montanitestudina hydei* (SQUCC 1517) and *Muritestudina chiangraiensis* (MFLUCC 17–2551) were selected as the outgroup taxa. Phylogenetic trees generated from maximum likelihood and Bayesian inference analyses were similar in overall topologies. The best-scoring RAxML tree with a final likelihood value of -22387.783323 is presented. The matrix had 1114 distinct alignment patterns, with 23.80% undetermined characters or gaps. Estimated base frequencies were as follows: A = 0.243359, 0.248863, G = 0.275982, T = 0.231796; substitution rates AC = 2.478083, AG = 4.357940, AT = 1.828936, CG = 1.187315, CT = 8.569946, GT = 1.000000. Tree-Length = 2.515173, gamma distribution shape parameter α = 0.171825. Support values for maximum likelihood (ML) above 70% and Bayesian posterior probabilities (PP) greater than 0.95 are given at the nodes.

**Figure 1.**
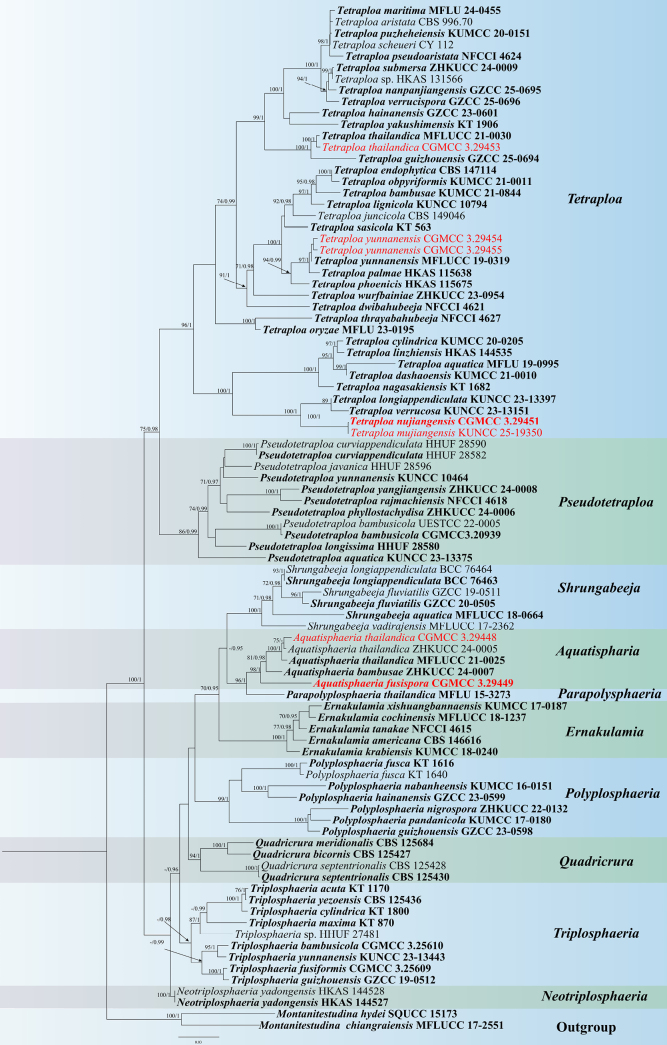
RAxML tree based on analysis of combined ITS, LSU, SSU, and *tub*2 dataset. RAxML bootstrap support values equal to or greater than 70% are given before the forward slash. Branches with Bayesian posterior probabilities equal to or higher than 0.95 are given after the forward slash. The tree is rooted to *Montanitestudina hydei* (SQUCC 15173) and *Muritestudina chiangraiensis* (MFLUCC 17–2551). The ex-type strains are in bold and the newly obtained sequences are indicated in red.

The multigene phylogenetic analyses showed that the seven fresh collections clustered within Tetraplosphaeriaceae (Fig. 1). Three known species, *Aquatisphaeria thailandica* (CGMCC 3.29448), *Tetraploa thailandica* (CGMCC 3.29453), and *T. yunnanensis* (CGMCC 3.29454 and CGMCC 3.29455) clustered with their ex-type strains, respectively. *Tetraploa nujiangensis* (CGMCC 3.29451 and KUNCC 25–19350) clustered sister to *T. longiappendiculata* (KUNCC 23–13397) and *T. verrucosa* (KUNCC 23–13151) in an independent clade. Similarly, *A. fusispora* (CGMCC 3.2944) was resolved as a distinct clade, sister to *A. bambusae* (ZHKUCC 24–0007), *A. thailandica* (MFLUCC 21–0025 and ZHKUCC 24–0005), and *Parapolyplosphaeria thailandica* (MFLU 15–3273).

### Taxonomy

#### Tetraploa

Taxon classificationFungiPleosporalesTetraplosphaeriaceae

Berk. & Broome

32876CE0-186C-5A77-8BA7-2E4A64D7E28D

##### Notes.

*Tetraploa* is the type genus of the family Tetraplosphaeriaceae (Dong et al. 2020; Hongsanan et al. 2020; Li et al. 2020, 2021; Tang et al. 2023; Liao et al. 2024). The genus was originally introduced by Berkeley and Broome (1850), with *T. aristata* designated as the type species. The asexual morph of *Tetraploa* is characterized by micronematous or no conidiophores, monoblastic conidiogenous cells, and tetraploate conidia composed of 4-euseptate, short-cylindrical, brown, vertical columns which are verrucose at the base, and with 4-setose, divergent, short or long septate appendages at the apex (Tanaka et al. 2009; Li et al. 2020; Liao et al. 2022, 2024). Species of *Tetraploa* occur as endophytes or saprobes on plant leaves and stems in both terrestrial and aquatic habitats (Ando 1992; Dong et al. 2020; Hyde et al. 2020; Li et al. 2021). They are commonly associated with bamboo and other herbaceous hosts (Phookamsak et al. 2022), as well as decaying wood (Liao et al. 2022; Nuñez Otaño et al. 2022; Kundu et al. 2024; Wang et al. 2024; Shen et al. 2025). According to Index Fungorum (accessed on March 2026), 52 species epithets are currently recognized in *Tetraploa*.

#### Tetraploa nujiangensis

Taxon classificationFungiPleosporalesTetraplosphaeriaceae

X.L. Ma, H.W. Shen & Z.L. Luo sp. nov.

5A328674-5DB2-5051-A598-CF70689F2B62

Fungal Names: FN 573531

Fig. 2

##### Etymology.

“*nujiangensis*” refers to the Nujiang River, where the species was collected.

**Figure 2.**
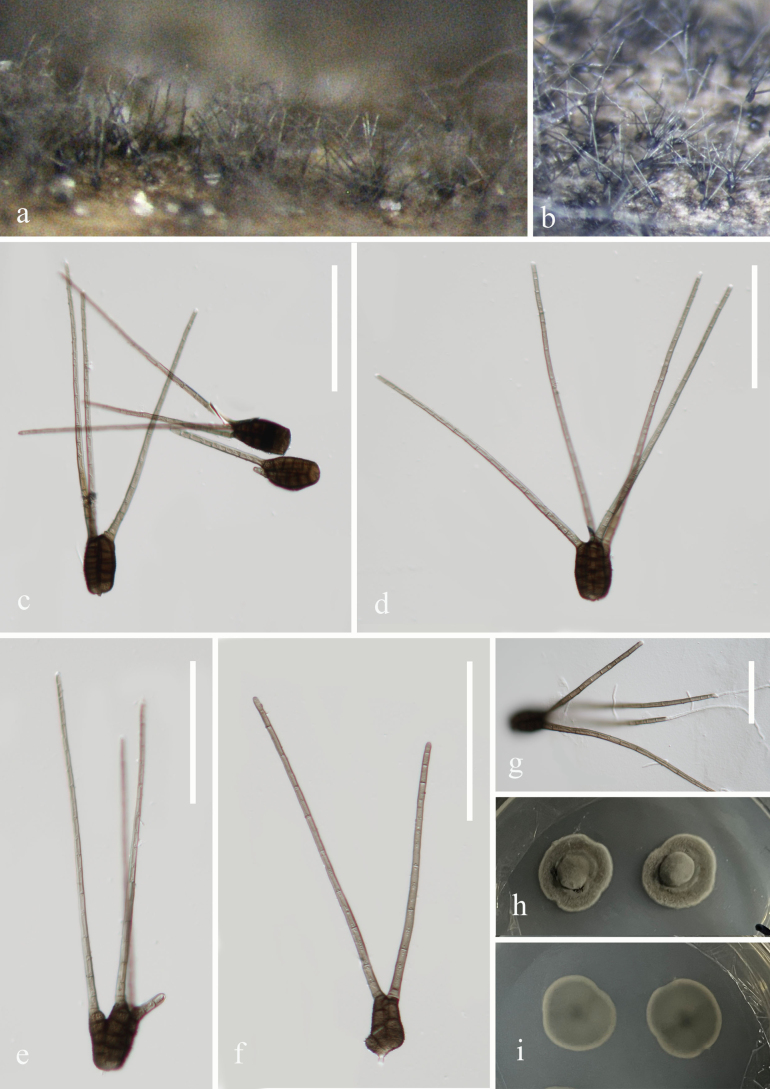
*Tetraploa nujiangensis* (HKAS 151662, holotype). **a, b**. Colonies on submerged decaying wood; **c–f**. Conidia; **g**. Germinating conidium; **h**. Colonies on PDA (front front); **i**. Colonies on PDA (front reverse). Scale bars: 100 µm (**c–f**); 50 µm (**g**).

##### Holotype.

HKAS 151662.

##### Description.

***Saprobic*** on submerged decaying wood. Sexual morph: undetermined. Asexual morph: hyphomycetous. ***Colonies*** effuse, dark brown to black, gregarious on host substrate. ***Mycelium*** partly immersed, branched, septate. ***Conidiophores*** absent. ***Conidiogenous cells*** monoblastic. ***Conidia*** 32–53 × 23–38 µm (x̄ = 45 × 29 µm, n = 30), solitary, unbranched, cylindrical with obtuse ends, brown, composed of four columns of cells, 3–6-septate in each column, verruculose, 2–4 appendages, mostly with four apical appendages. ***Appendages*** 136–262 µm long, 4–7 µm wide at the base, 1–2 µm wide at the apex, gradually tapering to the tip, divergent, pale brown to brown, 7–19-septate, straight or slightly flexuous, smooth-walled.

##### Culture characteristics.

Conidia germinate on water agar within 12 h, and germ tubes are produced from both ends. Colonies growing on PDA, and after 3 weeks of incubation at room temperature, the diameter reaches about 2 cm. Mycelia are dry, rough-surfaced, dense, superficial, raised, and spreading, with colony shape varying from circular to irregular and a fluffy texture. The upper surface of the colony is gray, while the reverse side is light gray with a white margin.

##### Material examined.

China • Yunnan Province, Nujiang River (26°90'57"N, 98°51'60"E), on submerged decaying wood, 25 February 2025, Wen-Peng Wang, S-7217 (HKAS 151662, holotype), ex-type CGMCC 3.29451 = KUNCC 25–19653; • *ibid*., 24°59'53"N, 98°51'49"E, on submerged decaying wood, 25 February 2025, Wen-Peng Wang, S-7252 (HKAS 151653, paratype), ex-paratype, KUNCC 25–19350.

##### Notes.

In our multi-locus phylogenetic analyses, *Tetraploa nujiangensis* is resolved as an independent lineage, sister to *T. longiappendiculata* (KUNCC 23–13397) and *T. verrucosa* (KUNCC 23–13151) with 100% ML and 1.00 PP (Fig. 1). Morphologically, *T. nujiangensis* resembles *T. longiappendiculata* and *T. verrucosa* in having cylindrical, verruculose conidia, composed of four columns of cells, and with 1–4 apical appendages (Tanaka et al. 2009; Wang et al. 2024; Shen et al. 2025). However, *T. nujiangensis* can be distinguished from *T. longiappendiculata* by its longer conidia (32–53 µm vs. 26–39 µm) and appendages (136–262 µm vs. 110–180 µm) (Wang et al. 2024), and from *T. verrucosa* by its longer appendages (136–262 µm vs. 78–179 µm) with more septa (7–19 vs. 3–10) appendages (Shen et al. 2025). The ITS sequence comparison shows that *T. nujiangensis* differs from *T. longiappendiculata* and *T. verrucosa* by 7% (414/447 bp) and 9% (425/467 bp), respectively; the LSU sequence differs by 1% (785/796 bp) and 1% (776/7787 bp), respectively, while the SSU sequence is identical to both species (100%). Based on both morphological characteristics and molecular evidence, we recognize *T. nujiangensis* as a new species of *Tetraploa*.

#### Tetraploa thailandica

Taxon classificationFungiPleosporalesTetraplosphaeriaceae

D.F. Bao, H Y. Su, K.D. Hyde & Z.L. Luo, Journal of Fungi 7 (8): 669 (2021)

C0E71536-7884-54F1-91D9-1CC7F2580BB9

Fungal Names: FN 558591

Fig. 3

##### Description.

***Saprobic*** on submerged decaying wood. Sexual morph: undetermined. Asexual morph: hyphomycetous. ***Colonies*** effuse and brown or dark greyish brown. ***Mycelium*** mostly immersed, composed of branched, septate, subhyaline hyphae. ***Conidiophores*** absent. ***Conidiogenous cells*** monoblastic. ***Conidia*** 29–44 × 20–31 µm (x̄ = 37 × 26 µm, n = 30) cylindrical, solitary, dry, septate, grayish-brown, pale brown to subhyaline at the apex when young, and brown to dark brown at maturity, verruculose and composed of 4 closely-adhered vertical columns of cells, each column 3–5-septate, with 2–4 brown to pale brown apical appendages. ***Appendages*** 19–138 µm long, 5–8 µm wide at the base, gradually tapering to the tip, 1–3 µm wide at the apex.

**Figure 3.**
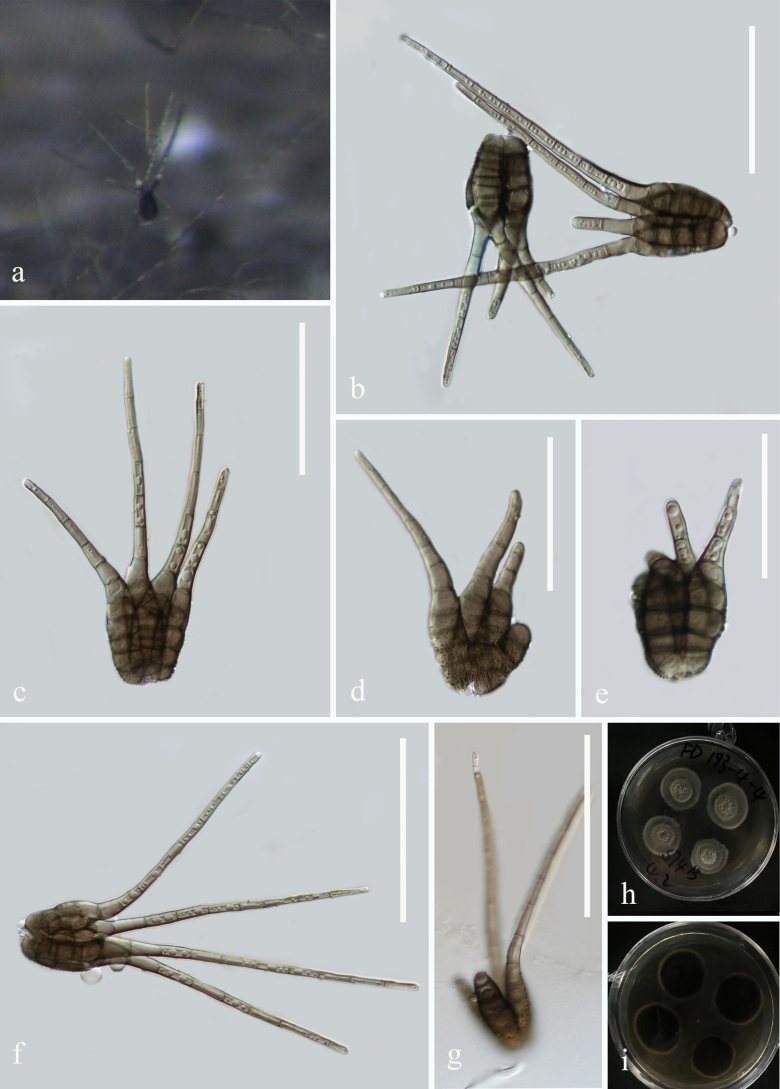
*Tetraploa thailandica* (HKAS 151652). **a**. Colonies on submerged decaying wood; **b–f**. Conidia; **g**. Germinating conidium; **h**. Colonies on PDA (front front); **i**. Colonies on PDA (front reverse). Scale bars: 40 µm (**e**); 60 µm (**b–d**); 80 µm (**f, g**).

##### Culture characteristics.

Conidia germinate on water agar within 12 h, and germ tubes produced from both ends. Colonies growing on PDA, and after 3 weeks of incubation at room temperature, the diameter reaches about 2 cm. Mycelia was superficial and circular with the entire margin flat and smooth. The central region of the upper surface is slightly lighter in color, appearing grayish brown, whereas the marginal region is darker, showing grayish green. The lower surface is dark brown to black, and no pigmentation is produced in the culture.

##### Material examined.

China • Yunnan Province, Nujiang River (25°14'12"N, 98°50'18"E), on submerged decaying wood, 25 February 2025, Wen-Peng Wang, S-7413(HKAS 151652), living culture, CGMCC 3.29453 = KUNCC 25–19660.

##### Notes.

In the phylogenetic analyses, our new isolate (KUNCC 25–19660) clustered with *Tetraploa thailandica* (MFLUCC 21–0030) with 100% ML and 1.00 PP (Fig. 1). Sequence comparisons revealed only three nucleotide differences in the ITS region and a single nucleotide difference in the LSU region between our isolate and the holotype. Morphologically, our new collection resembled the holotype of *T. thailandica* in having monoblastic polyblastic conidiogenous cells and cylindrical conidia composed of four columns of cells, and with 2–4 apical appendages (Bao et al. 2021). No significant morphological differences were observed. Based on the morphological and phylogenetic analyses, we therefore identified our new isolate as *T. thailandica*. This represents the first report of this species from China; previously, it was known only from freshwater habitats in Thailand.

#### Tetraploa yunnanensis

Taxon classificationFungiPleosporalesTetraplosphaeriaceae

W. Dong, H. Yang & H. Zhang, Fungal Diversity 105: 502 (2020)

48843002-CB09-5020-BFBA-658F4467E676

Fungal Names: FN 557937

Fig. 4

##### Description.

***Saprobic*** on submerged decaying wood. Sexual morph: undetermined. Asexual morph: hyphomycetous. ***Colonies*** effuse, dark brown to black, gregarious on host substrate. ***Mycelium*** partly immersed, branched, septate. ***Conidiophores*** absent. ***Conidiogenous cells*** monoblastic. ***Conidia*** 33–47 × 18–33 µm (x̄ = 39 × 25 µm, n = 30), solitary, unbranched, solitary, cylindrical, brown to dark brown, composed of 2–3 columns of cells, 4–6-septate in each column, verruculose, sometimes exhibiting basal separation between the cell columns, mostly with 2–3 apical appendages. ***Appendages*** 22–77 µm long, 5–10 µm wide at the base, gradually tapering to the tip, 1.5–4.5 µm wide at the apex, divergent, pale brown to brown, 1–7-septate, straight or slightly flexuous, smooth-walled.

**Figure 4.**
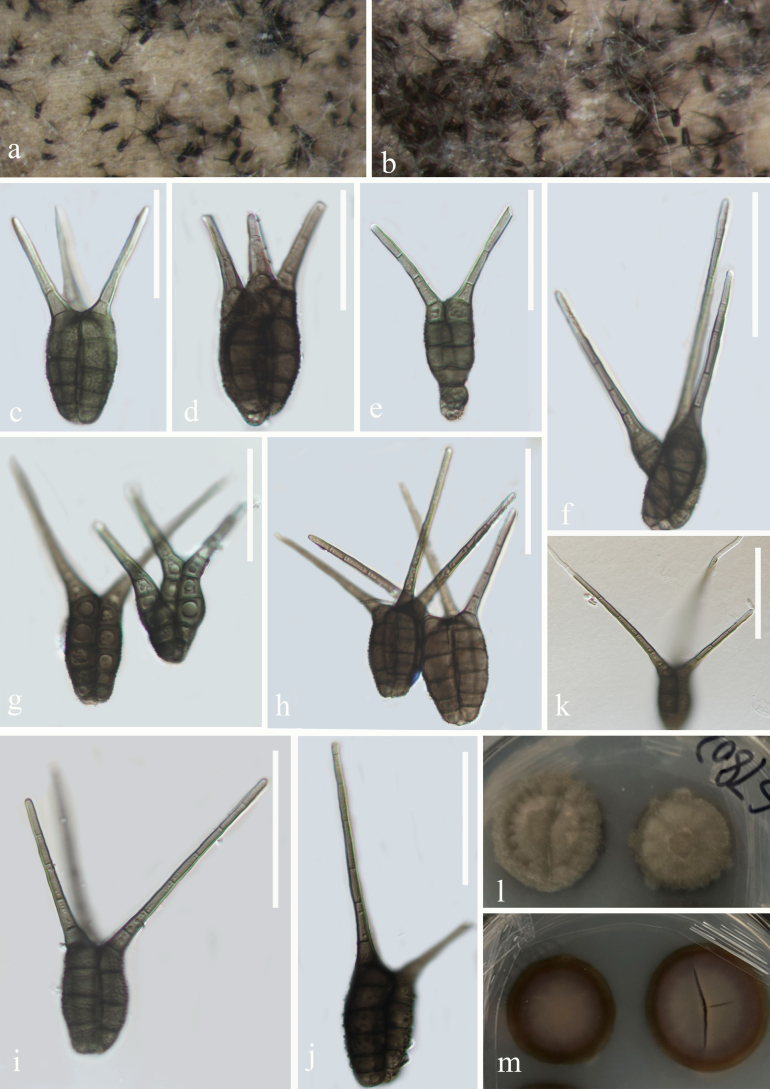
*Tetraploa yunnanensis* (HKAS 151661). **a, b**. Colonies on submerged decaying wood; **c–j**. Conidia; **k**. Germinating conidium; **l**. Colonies on PDA (front front); **m**. Colonies on PDA (front reverse). Scale bars: 40 µm (**c–e, g, h**); 50 µm (**f, i–k**).

##### Culture characteristics.

Conidia germinate on water agar within 12 h, and germ tubes produced from both ends. Colonies growing on PDA, and after 3 weeks of incubation at room temperature, the diameter reaches about 2 cm. It exhibits distinct textures on the surface, presenting fold-like structures and a fluffy texture. The reverse side is light brown, with an undulate margin that is brown to dark brown, and there are obvious cracks.

##### Material examined.

China • Yunnan Province, Nujiang River (24°23'60"N, 99°23'7"E), on submerged decaying wood, 23 February 2025, Hong-Wei Shen, S-7802 (HKAS 151661), living culture, CGMCC 3.29455; • *ibid*., 25°20'22"N, 98°51'19"E, on submerged decaying wood, 25 February 2025, Hong-Wei Shen, S-7774 (HKAS 151660), living culture, CGMCC 3.29454.

##### Notes.

In our phylogenetic analyses, the two new collections HKAS 151661 and HKAS 151660) clustered with the ex-type of *Tetraploa yunnanensis* (MFLUCC 19–0319) with 97% ML and 1.00 PP support (Fig. 1). Sequence analysis showed that there are no base differences in the ITS sequences between our new isolates and the type strain. Morphologically, our collections resemble *T. yunnanensis* in having monoblastic to polyblastic conidiogenous cells and cylindrical, verruculose conidia, composed of 2–3 columns of cells, and with 2–3 apical appendages (Dong et al. 2020). However, in our new collections, some conidia exhibited basal separation of the cell columns from one another, whereas this feature has not been reported in the holotype (Dong et al. 2020). This variation likely reflects phenotypic plasticity or intraspecific morphological variability influenced by environmental or substrate conditions, rather than a taxonomically significant distinction. Therefore, based on both morphological evidence and phylogenetic evidence, our new isolates are identified as *T. yunnanensis*.

#### Aquatisphaeria

Taxon classificationFungiPleosporalesTetraplosphaeriaceae

W.L. Li, N.G. Liu & Jian K. Liu, Phytotaxa 513(2): 122 (2021)

56579AAF-0414-5F7D-8D26-C32A24FECB22

##### Notes.

*Aquatisphaeria* was introduced by Li et al. (2021) with *A. thailandica* as the type species. The genus is characterized by macronematous, solitary, and unbranched conidiophores, monoblastic, determinate, and terminal conidiogenous cells, and subglobose or turbinate, muriform, dictyoseptate conidia with cylindrical appendages (Li et al. 2021; Zhao et al. 2024). Based on morphological and phylogenetic analyses, two species, *A. bambusae and A. thailandica*, are currently recognized in *Aquatisphaeria* (Index Fungorum, https://indexfungorum.org/, accessed on March 2026). Species in this genus have been reported from freshwater and terrestrial habitats in China and Thailand (Li et al. 2021; Zhao et al. 2024).

#### Aquatisphaeria thailandica

Taxon classificationFungiPleosporalesTetraplosphaeriaceae

W.L. Li, D.F. Bao & Jian K. Liu, Phytotaxa 513(2): 122 (2021)

7FF50FC7-A883-518D-953A-58FB5E5BE640

Fungal Names: FN 839208

Fig. 5

##### Description.

***Saprobic*** on submerged decaying wood in freshwater habitats. Sexual morph: undetermined. Asexual morph: hyphomycetous. ***Colonies*** on natural substrata are effuse, scattered, and dark brown to black. ***Mycelium*** partly immersed, partly superficial, septate, branched, smooth, sub-hyaline to light brown. ***Conidiophores*** 15–34 × 3–5 µm (x̄ = 24 × 4 µm, n = 15), macronematous, mononematous, solitary, cylindrical, curved at base, unbranched, septate, brown, smooth, thick-walled, and truncate at the apex. ***Conidiogenous cells*** 3–4 × 2–3 μm monoblastic, determinate, integrated, terminal, subcylindrical, brown. ***Conidia*** 41–64 × 37–62 µm (x̄ = 51 × 49 µm, n = 30), acrogenous, solitary, subglobose, thin-walled, internally composed of several columns of cells, pale brown when young, dark brown when mature, muriform, dictyoseptate, smooth-walled, with 4–7 (mostly 4) cylindrical, paler brown appendages arising from the conidial apex. ***Appendages*** 10–66 µm long, 3–6 µm wide at the base, gradually tapering to the tip, 1–3 µm wide at the apex, 1–6-septate.

**Figure 5.**
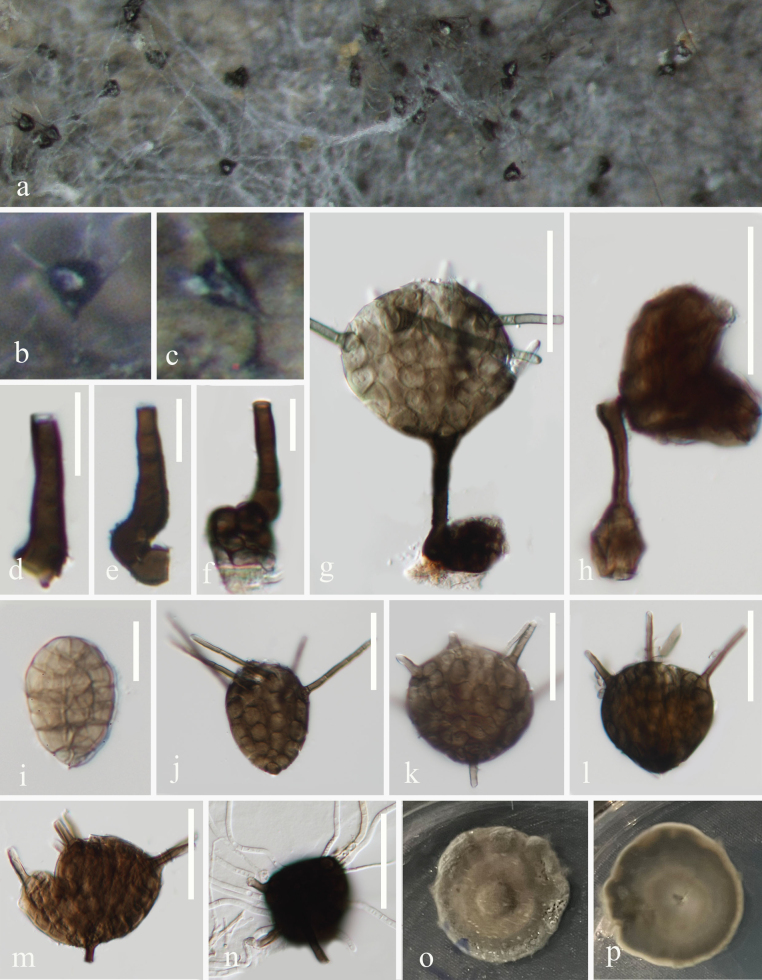
*Aquatisphaeria thailandica* (HKAS 151663). **a–c**. Colonies on submerged decaying wood; **d–f**. Conidiophores and Conidiogenous cells; **g, h**. Conidiophores and Conidia; **i–m**. Conidia; **n**. germinating conidium; **o**. Colonies on PDA (front front); **p**. Colonies on PDA (front reverse). Scale bars: 10 µm (**d–f, i**); 40 µm (**g, h, j–n**).

##### Culture characteristics.

Conidia germinate on water agar within 12 h, and germ tubes are produced from both ends. Colonies growing on PDA, and after 3 weeks of incubation at room temperature, the diameter reaches about 2 cm. Colonies on the medium are circular, floccose to velvety in texture. The surface is grayish white to grayish brown, rough, with dense aerial mycelia, dry in texture, and raised at the center. The colony margin is entire. The reverse is brownish to grayish brown, with conspicuous cracks.

##### Material examined.

China • Yunnan Province, Nujiang River (24°46'43"N, 98°54'45"E), on submerged decaying wood, 24 February 2025, Wen-Peng Wang, S-7361 (HKAS 151663), living culture, CGMCC 3.29448 = KUNCC 25–19407.

##### Notes.

In our phylogenetic analysis, the new isolate (KUNCC 25–19407) clustered with the *Aquatisphaeria thailandica* (MFLUCC 21–0025 and ZHKUCC 24–0005) with 100% ML and 1.00 PP support (Fig. 1). The new strain KUNCC 25–19407 closely resembles *A. thailandica* but can be distinguished from MFLUCC 21–0025 in having larger conidia (41–64 × 37–62 μm vs. 36–50 × 30–47 μm) and larger appendages (10–66 μm vs. 19–29 μm) (Li et al. 2021). It differs from ZHKUCC 24–0005 by possessing significantly larger conidia (41–64 × 37–62 μm vs. 18–38 × 15–25 μm) and larger appendages (10–66 μm vs. 11–20 × 2.5–4 μm) (Zhao et al. 2024). The ITS sequence comparison shows that KUNCC 25–19407 differs from MFLUCC 21–0025 and ZHKUCC 24–0005 by 2% (440/449 bp) and 1% (444/449 bp), respectively, whereas no differences were detected in LSU and SSU sequences. Therefore, based on both morphological similarity and phylogenetic affinity, our new isolate is identified as *A. thailandica*.

#### Aquatisphaeria fusispora

Taxon classificationFungiPleosporalesTetraplosphaeriaceae

X.L. Ma, H.W. Shen & Z.L. Luo sp. nov.

2B4139E4-8666-5EB4-B6D1-D7AC4CAA4054

Fungal Names: FN 573532

Fig. 6

##### Etymology.

The specific epithet “*fusispora*” refers to the fusiform shape of the conidia observed in this species.

**Figure 6.**
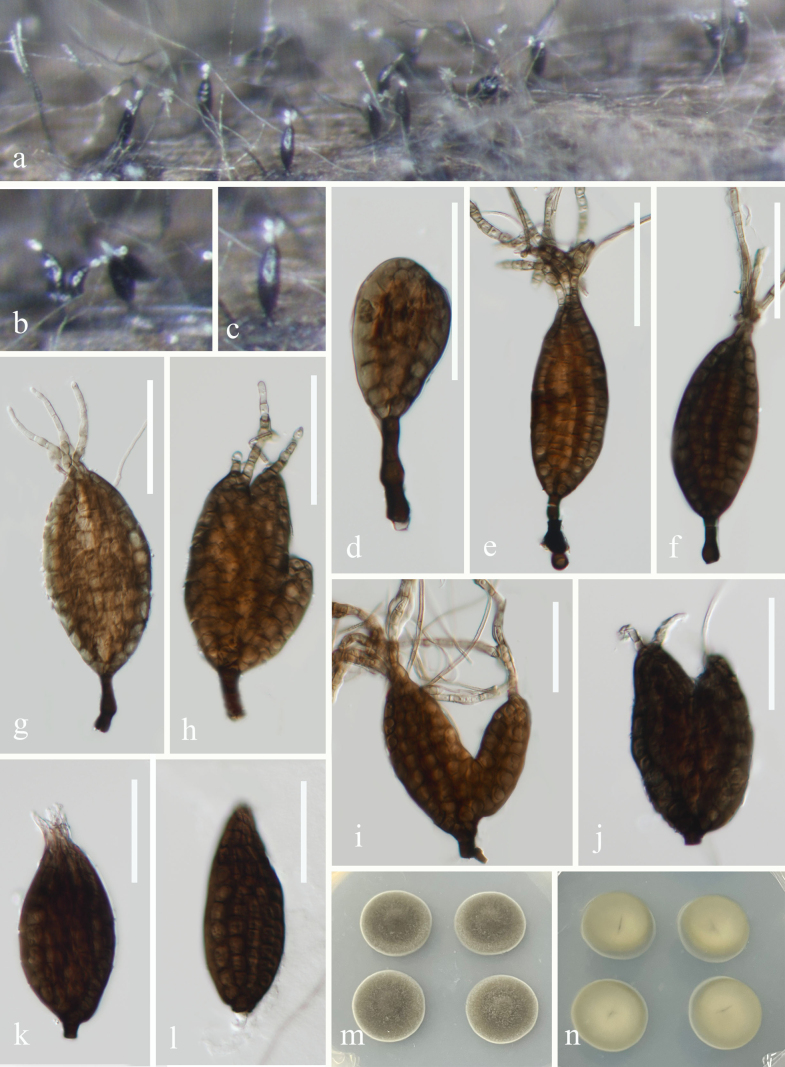
*Aquatisphaeria fusispora* (HKAS 151664, holotype). **a–c**. Colonies on submerged decaying wood; **d–h**. Conidiophores and Conidia; **i–k**. Conidia; **l**. Germinating conidium; **m**. Colonies on PDA (front front); **n**. Colonies on PDA (front reverse). Scale bars: 50 µm (**d–h**); 40 µm (**i–l**).

##### Holotype.

HKAS 151664.

##### Description.

***Saprobic*** on submerged decaying wood in freshwater habitats. Sexual morph: undetermined. Asexual morph: hyphomycetous. ***Mycelium*** partly immersed and partly superficial, septate, branched, smooth-walled, subhyaline to pale brown. ***Conidiophores*** 17–33 × 5–9 μm (x̄= 22 × 7 μm, n = 15), macronematous, mononematous, solitary, cylindrical, erect to slightly curved, unbranched, 2–4-septate, brown to dark brown, smooth, thick-walled, with truncate apex. ***Conidiogenous cells*** 6.8–7.9 × 4.0–5.7 μm, monoblastic, terminal, integrated, cylindrical, brown to dark brown, sometimes percurrently proliferating. ***Conidia*** 47–97 × 22–50 μm (x̄ = 76 × 32 μm, n = 30), acrogenous, solitary, initially obovoid, becoming ellipsoidal to fusiform at maturity, composed of a basal cell and multiple columns of cells, sometimes divided into 2–3 separate columns from the base or middle part, smooth, thin-walled, with 2–5 apical appendages; ***basal cell*** cylindrical, truncate, black or dark brown, 6–8 × 4–6 µm; ***column*** cylindrical, brown to dark brown, 9–14-septate, 63–95 μm long and 4–8 μm wide (excluding appendages); ***appendages*** unbranched, hyphae-like, 5–158 μm long, 1–3 μm wide at the apex, 3–6 μm wide at the base, pale brown to brown, 1–18-septate, sometimes surrounded by a gelatinous sheath at the apex.

##### Culture characteristics.

Conidia germinate on water agar within 12 h, and germ tubes are produced from both ends. Colonies growing on PDA, and after 3 weeks of incubation at room temperature, the diameter reaches about 1.5 cm. Colonies on the medium are circular, floccose to velvety in texture, circular, green on both surface and reverse, with dense aerial mycelia and a velvety texture; margins entire.

##### Material examined.

China • Yunnan Province, Nujiang River (25°39'7"N, 98°53'8"E), on submerged decaying wood, 25 February 2025, Hong-Wei Shen, S-7780 (HKAS 151664, holotype), ex-type CGMCC 3.29449.

##### Notes.

Phylogenetic analyses showed that *Aquatisphaeria fusispora* clustered with *A. bambusae* and *A. thailandica* and *Parapolyplosphaeria thailandica* with 96% ML and 1.00 PP support (Fig. 1). Morphologically, *A. fusispora* resembles these closely related species in having solitary, unbranched, cylindrical conidiophores, and conidia with appendage (Li et al. 2021; Tang et al. 2023; Zhao et al. 2024; Manawasinghe et al. 2025). However, *A. fusispora* can be distinguished from *A. bambusae* and *A. thailandica* by ellipsoidal conidia, composed of a basal cell, sometimes divided into 2–3 separate columns from the base or middle part, and the apical appendage may be surrounded by a gelatinous sheath (Li et al. 2021; Tang et al. 2023; Zhao et al. 2024). In contrast, the conidia of *A. bambusae* and *A. thailandica* are globose and the columns of conidia cells not separating, and without any gelatinous sheath at the apex of appendage (Li et al. 2021; Tang et al. 2023; Zhao et al. 2024). Furthermore, *A. fusispora* is distinguished from *Parapolyplosphaeria thailandica* which produces muriform, globose, obovoid, pyriform to ellipsoidal conidia, and lacking any columnar structure, and the apical appendages are without any mucilaginous sheath (Manawasinghe et al. 2025). Based on the distinct morphological characters and clear phylogenetic divergence, *Aquatisphaeria furcata* is therefore introduced as a new species.

#### Aquatisphaeria compacta

Taxon classificationFungiPleosporalesTetraplosphaeriaceae

(R.F. Castañeda, Guarro & Cano) X.L. Ma, H.W. Shen & Z.L. Luo comb. nov.

E0422E8B-CDAC-55D8-911A-4826976AA609

Fungal Names: FN 573533

##### Basionym.

*Ceratosporella compacta* R.F. Castañeda, Guarro & Cano, Mycotaxon 60: 276 (1996).

##### Holotype.

Cuba: Viñales, Pinar del Río, on fallen decaying stem of unidentified member of the Poaceae, November 1994, R. F. Castañeda (INIFAT C94/162).

##### Notes.

*Ceratosporella compacta* was introduced by Castañeda et al. (1996) from terrestrial decaying plant material in Cuba. The species is characterized by mononematosa, eramosa, erecta conidiophores; and monoblastic terminal, cylindrical conidiogenous cells, and cheirosporous, acrogenous, solitary conidia, composed of a basal cell and 4–5 vertical columns of cells, with rounded apical appendages surrounded by a gelatinous sheath (Castañeda et al. 1996). These characteristics are highly similar to those of *Aquatisphaeria fusispora*, but the column of *C. compacta* is smaller than *A. fusispora* (65–70 × 4–5 µm vs. 63–95 × 4–8 µm) and with shorter appendages (15–46 µm vs. 5–158 µm). Therefore, based on highly similar morphological characteristics, we transferred *C. compacta* to *Aquatisphaeria* as *A. compacta*.

## Discussion

This study provides new insights into the taxonomy and phylogeny of *Tetraploa* and *Aquatisphaeria* (Tetraplosphaeriaceae, Pleosporales) based on collections from freshwater habitats in the Nujiang River Basin, Yunnan, China. Five taxa were identified from submerged woody substrates, including two new species (*Tetraploa nujiangensis* and *Aquatisphaeria fusispora*), one new record (*T. thailandica*), and two known species (*A. thailandica* and *T. yunnanensis*). The discovery of multiple *Tetraploa* and *Aquatisphaeria* species from a single freshwater ecosystem significantly enriches our understanding of the diversity, ecological adaptation, and evolutionary differentiation of Tetraplosphaeriaceae.

Morphologically, *A. fusispora* is clearly distinguishable from the two previously described species of the genus, *A. bambusae* and *A. thailandica*, both of which are characterized by solitary, unbranched, cylindrical conidiophores and subglobose conidia bearing appendages (Li et al. 2021; Zhao et al. 2024). In contrast, *A. fusispora* possesses significantly larger conidia that are ellipsoidal to fusiform in shape, composed of a basal cell and 3–8 cellular columns, which are sometimes forked at the base or middle, and bear long, hyphae-like apical appendages. These characters represent a marked degree of morphological differentiation within *Aquatisphaeria*. However, the species is currently known from a single specimen, and thus the full extent of its morphological variation cannot yet be adequately assessed. Despite the pronounced morphological differences, multigene phylogenetic analyses consistently place *A. fusispora* within a single, strongly supported clade together with *A. bambusae*, *A. thailandica*, and *P. thailandica* (Fig. 1). Within this clade, *A. fusispora* does not form a deeply divergent or phylogenetically isolated lineage. Therefore, there is insufficient evidence to justify the establishment of a new genus to accommodate *A. fusispora*. Instead, based on the combined morphological and phylogenetic evidence, we herein describe *A. fusispora* as a new species within *Aquatisphaeria*. Given the limited taxon sampling, both in terms of available specimens and molecular data, the apparent incongruence between morphological traits and phylogenetic relationships should be interpreted with caution. Additional collections and more comprehensive phylogenetic analyses will be necessary to further clarify the taxonomic placement of *A. fusispora* and to better elucidate species boundaries and evolutionary relationships within *Aquatisphaeria*, a genus that remains poorly represented in current taxonomic frameworks.

All taxa discovered in this study were isolated from submerged woody substrates, emphasizing a pronounced ecological preference for lignicolous habitats within freshwater environments. This substrate affinity aligns with previous reports that Tetraploa-like fungi frequently colonize submerged bamboo, woody debris, or leaf litter in both terrestrial and aquatic ecosystems (Tanaka et al. 2009; Phookamsak et al. 2022; Zhao et al. 2024; Manawasinghe et al. 2025). The consistent occurrence of Tetraplosphaeriaceae on decaying wood suggests a saprobic lifestyle relying on lignocellulose degradation, an ecological role fundamental to nutrient recycling in freshwater ecosystems. Adaptation to aquatic habitats likely involves morphological and physiological modifications, particularly the evolution of appendaged conidia that facilitate spore attachment, dispersal, or flotation. Such structures enhance colonization success in flowing waters, representing convergent adaptations to aquatic conditions observed across diverse Ascomycetes (Gareth Jones 2006; Shearer et al. 2009; Calabon et al. 2022, 2023). The shared presence of appendaged conidia among all five taxa from the Nujiang River Basin underscores the evolutionary significance of these traits under water-mediated selective pressures.

From a biogeographical perspective, the discovery of these taxa from the Nujiang River Basin extends the known distributional range of Tetraplosphaeriaceae. This family was initially documented from bamboo and wood substrates in tropical and subtropical Asia, particularly Japan and India (Rifai et al. 1988; Pratibha and Bhat 2008; Tanaka et al. 2009), but its freshwater members remain poorly explored (Hyde et al. 2016; Dong et al. 2020). The identification of *T. thailandica* as a new record for China expands its known range northward, suggesting strong biogeographical connectivity between Yunnan and Southeast Asia (Bao et al. 2021; Li et al. 2021; Zhao et al. 2024). The Nujiang River, which flows southward into the Salween Basin, may serve as a dispersal corridor facilitating fungal migration and genetic exchange across regional freshwater systems. The coexistence of multiple Tetraplosphaeriaceae species within a single basin also reflects the high ecological heterogeneity and microhabitat diversity characteristic of Yunnan’s riverine ecosystems (Luo et al. 2018; Shen et al. 2022; Wang et al. 2024). Moreover, the discovery of two novel species and one new record from a single river system underscores the immense but still underestimated fungal diversity in subtropical freshwater environments. Future research integrating molecular phylogenetics, ecological characterization, and functional analyses will be essential to elucidate the evolutionary history, dispersal mechanisms, and ecological roles of Tetraplosphaeriaceae and related aquatic fungi in global freshwater ecosystems.

## Supplementary Material

XML Treatment for Tetraploa

XML Treatment for Tetraploa nujiangensis

XML Treatment for Tetraploa thailandica

XML Treatment for Tetraploa yunnanensis

XML Treatment for Aquatisphaeria

XML Treatment for Aquatisphaeria thailandica

XML Treatment for Aquatisphaeria fusispora

XML Treatment for Aquatisphaeria compacta

## Additional information

### Artificial Intelligence (AI) use

The authors accept full responsibility for the content of the manuscript, including the disclosure of any use of AI.

No AI tools were used in the preparation of this manuscript.

### Author ORCIDs

X.-L. Ma https://orcid.org/0009-0007-3348-5496

M.-J. Zhu https://orcid.org/0009-0009-0482-5185

F.-X. Ma https://orcid.org/0009-0008-0184-0999

Z.-L. Luo https://orcid.org/0000-0001-7307-4885

H.-W. Shen https://orcid.org/0000-0003-2508-1970

### Data availability

All of the data that support the findings of this study are available in the main text.
